# Mormyrid fish as models for investigating sensory‐motor integration: A behavioural perspective

**DOI:** 10.1111/jzo.13046

**Published:** 2023-01-30

**Authors:** S. Skeels, G. von der Emde, T. Burt de Perera

**Affiliations:** ^1^ Department of Biology University of Oxford Oxford UK; ^2^ Institute of Zoology University of Bonn Bonn Germany

**Keywords:** weakly electric fish, mormyrids, *Gnathonemus petersii*, sensory‐motor integration, behaviour, active electrolocation, object recognition

## Abstract

Animals possess senses which gather information from their environment. They can tune into important aspects of this information and decide on the most appropriate response, requiring coordination of their sensory and motor systems. This interaction is bidirectional. Animals can actively shape their perception with self‐driven motion, altering sensory flow to maximise the environmental information they are able to extract. Mormyrid fish are excellent candidates for studying sensory‐motor interactions, because they possess a unique sensory system (the active electric sense) and exhibit notable behaviours that seem to be associated with electrosensing. This review will take a behavioural approach to unpicking this relationship, using active electrolocation as an example where body movements and sensing capabilities are highly related and can be assessed in tandem. Active electrolocation is the process where individuals will generate and detect low‐voltage electric fields to locate and recognise nearby objects. We will focus on research in the mormyrid *Gnathonemus petersii* (*G. petersii*), given the extensive study of this species, particularly its object recognition abilities. By studying object detection and recognition, we can assess the potential benefits of self‐driven movements to enhance selection of biologically relevant information. Finally, these findings are highly relevant to understanding the involvement of movement in shaping the sensory experience of animals that use other sensory modalities. Understanding the overlap between sensory and motor systems will give insight into how different species have become adapted to their environments.

## Graphical abstract

We demonstrate the value of taking a behavioural approach to understanding sensory‐motor integration using mormyrid fish as models. These fish have a well‐characterised active electric sense and show locomotor behaviours that seem to be closely coupled with electrosensing, and these can be studied simultaneously during active electrolocation. We show that these components can be interrogated by carefully designed behavioural experiments and insight from these fish can be applied to understanding active sensing more generally.

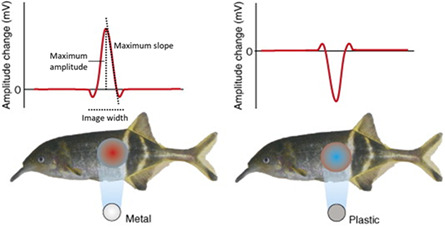

## Introduction

How sensory and motor systems operate together is an important and interesting area of research, since it helps us to understand and interpret animal behaviour. Sensory systems capture information from the environment which help an individual to determine an appropriate action for a particular situation, and, in turn, their own body movements can sculpt the information detected by these sensing systems (reviewed by Hofmann, Sanguinetti‐Scheck, Künzel, et al., 2013). The role of eye and head movements in visual perception has been well‐studied in a number of taxa (see reviews: Kral, 2003; Land, 1999). For example, mice will alter their head position and head movements when completing a jumping task under monocular conditions, potentially to facilitate distance estimation via position or motion parallax (Parker et al., 2022). As such, sensory and motor systems can be thought of in a loop framework, each system acting as an input for the other. This dynamic interaction allows animals to tailor their responses in real time which has adaptive advantages. It is important for an individual to identify and categorise signals correctly, as a mistake could have consequences on an individual's survival or reproductive success. For example, mistaking a predator for a conspecific could lead to serious injury or death if courtship behaviours are triggered rather than defensive or escape behaviours. This review aims to illustrate how one group of weakly electric fish, the African Mormyridae, are well‐suited for studying this problem, which has wide applicability to other animals and sensory systems.

Active sensing and active sensing strategies are commonly used for studying sensory‐motor interactions. In this review, we define active sensing in general terms as the process by which an animal uses self‐generated energy to investigate their surroundings (Hofmann, Sanguinetti‐Scheck, Künzel, et al., 2013; Nelson & MacIver, 2006; Zweifel & Hartmann, 2020). Examples include echolocation in bats (Griffin, 1944; Metzner & Müller, 2016) and active electrolocation in fish (Bennett, 1970; Caputi & Budelli, 2006; von der Emde, 2006).

Active sensing strategies are the processes by which sensory flow is modified by movement (Hofmann, Sanguinetti‐Scheck, Künzel, et al., 2013; Parker et al., 2022). Movements can be isolated to the sensor/emitter of the signal, or be more wide ranging, consisting of whole‐body movements (Hofmann, Sanguinetti‐Scheck, Künzel, et al., 2013; Kral, 2003). These strategies are utilised by both active and passive sensory systems, the latter of which relies on external energy not controlled by the animal (Hofmann, Sanguinetti‐Scheck, Künzel, et al., 2013; Nelson & MacIver, 2006). As such, senses which are considered passive (e.g. vision) can often be used in an active way (for a discussion on active vision see Parker et al., 2022). Active sensing strategies have been particularly well‐studied in echolocating bats. Echolocation is where an animal will produce a series of high‐frequency acoustic calls and then listen to their echoes as they bounce off objects within the environment (Surlykke et al., 2009). The information from these echoes can be used to locate prey and to navigate around obstacles (Surlykke et al., 2009). Research has been undertaken on how bats can modify the information they get from this sensory modality, by either moving body parts associated with call production or detection. For example, *Myotis daubentonii* are thought to change the aperture of their mouths to control their ‘acoustic gaze’, with a wider mouth allowing for a louder, narrower echolocation beam, meaning that they can be more directional with their calls (Surlykke et al., 2009). Echolocating porpoises can also adjust their ‘acoustic gaze’ depending on the environmental context (Wisniewska et al., 2012). Additionally, rhinolophid and hipposiderid bats will move their ears quickly which seems to allow them to encode the direction of their target (usually prey) in the form of time‐frequency Doppler signatures (Yin & Müller, 2019).

The active sensing strategies we have described so far have focussed on movements of the emitter or sensor; however, sometimes whole‐body movements are involved (Kral, 2003). Optic flow is a phenomenon observed in a wide range of taxa, including insects (e.g. Egelhaaf et al., 2014; Srinivasan et al., 2000), fish (Karlsson et al., 2022; Sibeaux et al., 2022) and bats (e.g. Kugler et al., 2019). During optic flow, egocentric movements cause visual images to shift across the retina, and changes in how these images are projected onto the retina provide an animal with precise information about the layout of their environment, thereby allowing them to navigate and forage in complex environments effectively (Egelhaaf et al., 2014; Karlsson et al., 2022; Kugler et al., 2019; Sibeaux et al., 2022; Srinivasan et al., 2000). Another example of how egocentric motion can create sensory flow is seen in blind cave fish. They have been shown to use the water movement as they swim past an object to obtain information about its identity and location through their lateral line‐ known as hydrodynamic imaging (Burt de Perera, 2004; von Campenhausen et al., 1981; Weissert & von Campenhausen, 1981; reviewed by Windsor, 2014). In sum, movements can be used in a range of ways to optimise perception of the environment (e.g. Hofmann, Sanguinetti‐Scheck, Künzel, et al., 2013; Parker et al., 2022).

Weakly electric fish are excellent candidates for examining sensory‐motor interactions, because they have a well‐characterised electrosensory system and show locomotive behaviours associated with electrosensing (e.g. Engelmann et al., 2021; Hofmann, Sanguinetti‐Scheck, Künzel, et al., 2013; Sawtell et al., 2005; Toerring & Belbenoit, 1979; Toerring & Moller, 1984; von der Emde, 2006). These components can be studied in tandem which is important as they do not work in isolation but as a coordinated unit through feedback (Clarke & Maler, 2017; Hofmann, Sanguinetti‐Scheck, Künzel, et al., 2013). Sensory and motor activity can also be quantified, meaning that we can assess how one system impacts the other in an objective way (e.g. Hofmann et al., 2014; Pedraja et al., 2018, 2020). As such, these animals fit within the framework of Krogh's principle, which states that for every problem, there is an animal well‐suited to solving it (Krogh, 1929); in this case, these fish provide us the opportunity to develop a comprehensive understanding of how sensory and motor systems coordinate their activity and optimise behaviour (see review on linking active sensing with spatial learning in these fish: Engelmann et al., 2021).

This review will take a behavioural approach to unravel the relationship between sensory and motor systems in these animals. We believe that studying behaviour in an experimental setting is an informative way of tackling this question (Pearce, 2008). We can carefully design experiments that change the sensory input received by an animal and then record its motor output (behaviour), from here we can make neural inferences on how these streams might be processed and integrated together (e.g. Schumacher, Burt de Perera, Thenert, et al., 2016; Sibeaux et al., 2022; Skeels et al., 2022). These experiments have the advantage that they can be run with little/no physical manipulation of the animal, minimising the risk of unintended effects (Animal Behaviour, 2018; Parker et al., 2022; Schumacher, Burt de Perera, Thenert, et al., 2016). As a consequence, the behaviours observed in response to a stimulus are likely to be more naturalistic, meaning we can be more confident in the conclusions we draw when describing and interpreting the behaviour (Parker et al., 2022).

It is worth acknowledging that behaviours recorded in a laboratory setting can deviate from what is observed in nature (Henninger et al., 2018). There is still great value in conducting behavioural experiments within this setting however, particularly for answering questions which would be difficult to do in a natural (uncontrolled) environment, such as our question of untangling the relationship between sensory and motor systems, where there is a need to monitor sensory and motor activity precisely in real time (e.g. Hofmann et al., 2014, 2017; Hofmann, Sanguinetti‐Scheck, Gómez‐Sena, et al., 2013; Pedraja et al., 2018, 2020). Technological developments, such as wireless electrophysiology (e.g. Vinepinsky et al., 2017), long‐term behavioural tracking (e.g. Jun et al., 2014) and machine learning (e.g. Pedraja et al., 2021) have improved the interpretation of behaviour in laboratory conditions while reducing the need for more invasive measures. Finally, it makes sense to take a behavioural approach given that evolution ultimately acts on behaviour (see these works for discussions of behaviour in an evolutionary context: Piaget, 2006; Slater & Halliday, 1994; Stevens, 2013). For example, diversification in communication signalling in these animals has promoted speciation (Carlson et al., 2011). This review will therefore use a behavioural perspective to discuss how one group of weakly electric fish (mormyrids) can be used as models for understanding sensory‐motor integration.

## Weakly electric fish: A brief background

Weakly electric fish have evolved the ability to generate and detect low voltage fields in the environment which they can use for localisation—commonly referred to as ‘active electrolocation’ (Heiligenberg, 1973; Lissmann, 1951; Lissmann, 1958; Lissmann & Machin, 1958; reviewed by von der Emde, 2006; von der Emde et al., 2008). They can also communciate with conspecifics using their electric signals, which is known as ‘electrocommunication’ (Gebhardt et al., 2012; Moller & Bauer, 1973; Worm et al., 2017). There are two independent lineages of weakly electric fish: the Gymnotiformes from the Neotropics (including South America) and the Mormyriformes (Mormyroidae) from Africa (Bullock et al., 1983; Winemiller & Adite, 1997). Both lineages developed these abilities at a similar time to one another, around 100 million years (Lavoué et al., 2012). Within the gymnotiformes, fish generate their electric fields by producing either pulse or wave‐type electric organ discharges, EODs, whereas, the vast majority of mormyriformes produce pulse‐type EODs (reviewed by Sawtell et al., 2005). Pulse‐type fish emit short electric pulses separated by periods of silence much longer than the pulses themselves, and will increase their discharge rate depending on the environmental context (Bennett, 1970; Sawtell et al., 2005). During electrocommunication, the fish encode information according to the timing of these events (Sawtell et al., 2005). Conversely, wave‐type fish produce quasi‐sinusoidal EODs at frequencies up to 1800 Hz (Sawtell et al., 2005) and change their discharge rate relatively little (Bennett, 1970). However, they will alter their signal frequency if they encounter a frequency similar to their own to avoid interference (Watanabe & Takeda, 1963). In both wave‐type and pulse‐type fish, objects within the electric field will cause amplitude modulations of the signal, with the spatial and frequency characteristics of these modulations being dependent on the stimulus (Sawtell et al., 2005). In this review, we will be limiting our focus to mormyriformes, in part because to cover both lineages in sufficient detail within a single review would be difficult, but more importantly, because of the extensive research that has been done in one family (Mormyridae) to link behaviour to sensing (e.g. Hofmann et al., 2014, 2017; Hofmann, Sanguinetti‐Scheck, Gómez‐Sena, et al., 2013; Pedraja et al., 2020; Schumacher, Burt de Perera, & von der Emde, 2016; Toerring & Belbenoit, 1979; Toerring & Moller, 1984; von der Emde & Zeymer, 2020).

## *Gnathonemus petersii* is an excellent model for studying sensory‐motor interactions

*Gnathonemus petersii* (*G. petersii*) is the main mormyrid species we will be discussing in the context of sensory‐motor integration. It is an excellent candidate species, given how much we known about its active electrosensory system and movement patterns during object sensing (e.g. Engelmann et al., 2008; Toerring & Belbenoit, 1979; Toerring & Moller, 1984; von der Emde, 2006; von der Emde et al., 2008).

**Figure 1:**
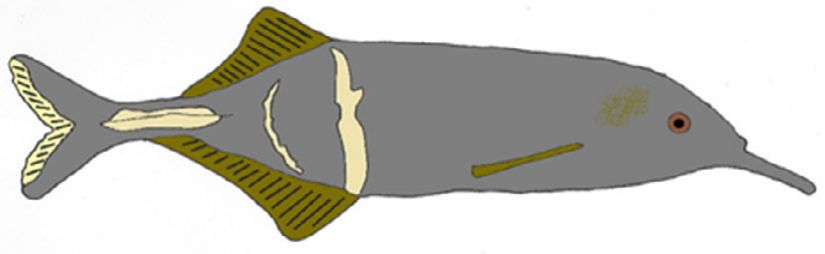
Illustration of the mormyrid, *Gnathonemus* petersii. They have a striking appearance, which includes an enlarged anal fin, distinctive stripes on the lower flank and a movable chin appendage (Schnauzenorgan).

*Gnathonemus petersii* (Günther 1862) is a mormyrid species found in turbid rivers and lakes across Central and Western Africa (Moller et al., 1979; Olaosebikan et al., 2020; Fig. 1). These fish are nocturnal and will travel away from their daytime rest sites to forage for small invertebrates hidden in the substrate (Corbet, 1961; Moller et al., 1979; von der Emde & Bleckmann, 1998). Their habitats typically experience rainy and dry seasons (Wuraola & Adetola, 2011), so individuals tend to migrate between different areas over the course of the year, for example, they tend to breed during the rainy season in flooded plains (Okedi, 1969 cited in Cain et al., 1994; Landsman, 1991). Like other mormyrids, it is likely that they are predated upon by larger fish, such as electrosensing catfish (Hanika & Kramer, 1999, 2000). As *G. petersii* live in poorly lit and sediment‐filled environments, they are heavily reliant on their active electrosensory system for foraging (e.g. von der Emde & Bleckmann, 1998; von der Emde & Zeymer, 2020), communication (e.g. Pedraja et al., 2021) and spatial navigation (e.g. Jung et al., 2019; Schumacher, von der Emde, et al., 2017). The importance of the active electric sense to everyday life in these animals, the amount of documentation on how their active electric sense works, and their locomotor patterns (*more on this later*) makes *G. petersii* an ideal candidate for understanding how these systems coordinate activity, and enable them to behave adaptively.

## Studying mormyrids during active electrolocation gives insight into sensory‐motor integration

Active electrolocation (object detection with the active electric sense) provides us with a unique opportunity to investigate and assess the relationship between sensory and motor systems given both have been characterised to a degree and they are quantifiable.

**Figure 2:**
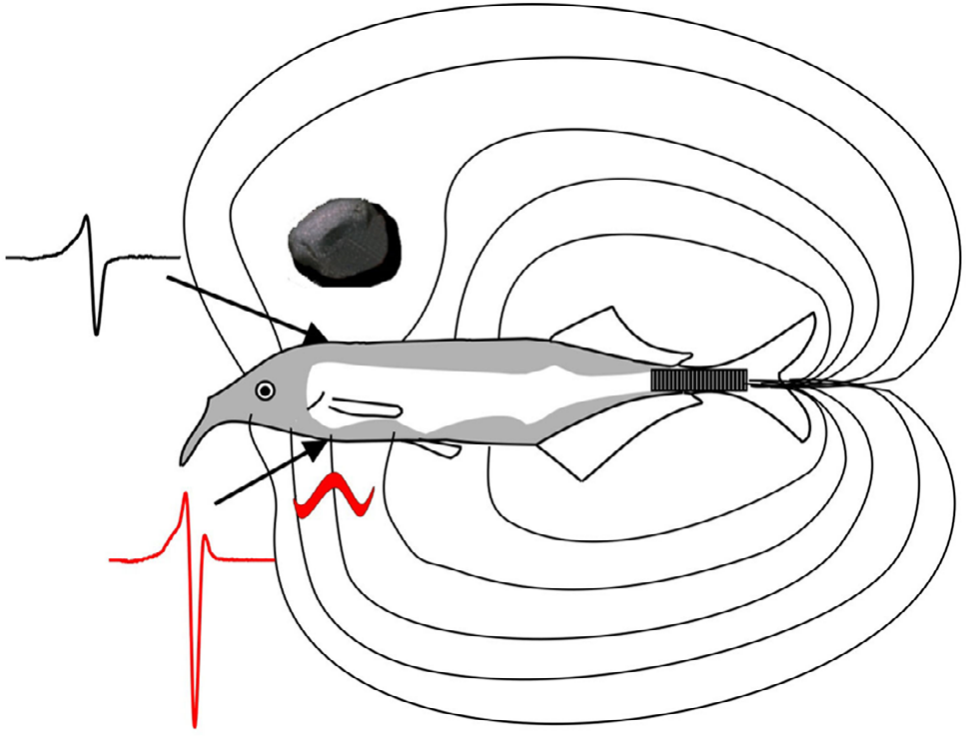
Schematic of *G. petersii's* active electrosensory system. EODs are produced by the electric organ in the caudal peduncle (black bar) to form electric fields around the animal. The field and local EODs are modified by nearby objects. Modification depends on the properties of the object. The worm (shown in red) is a conductor so increases field line density, and increases the amplitude of local EODs in addition to distorting their waveforms. Conversely, the stone (shown in black) is a non‐conductor so field line density decreases. At the same time, the amplitude of local EODs decreases but their waveforms remain the same. These object‐induced changes are then detected by specialised receptors on the skin (shown in grey). Diagram from von der Emde (2006).

**Figure 3:**
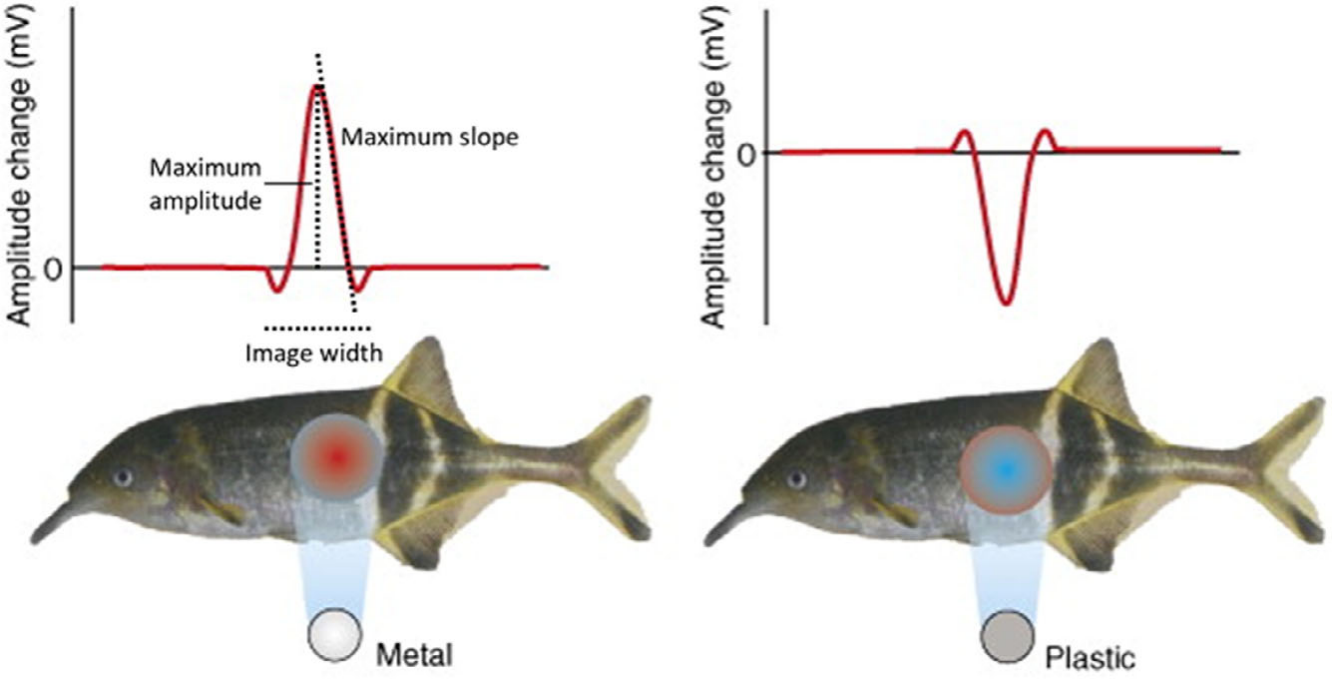
Objects projecting electric images onto the flanks of *G. petersii*. The metal object (shown on the left) projects an image on the skin which consists of a central region where there is an increase in local EOD amplitude, shown in red, followed by a surrounding region where local amplitude decreases, shown in blue. The plastic object (shown on the right) evokes the opposite pattern. Both electric images have the characteristic ‘Mexican Hat’ profile when local EOD amplitude change is plotted against position along the midline of the body (shown above the two fish). The dotted lines indicate three key image parameters from a static electric image that can be used to encode object properties (von der Emde, 2006). Adapted from von der Emde (2010).

The active electric sense is *G. petersii's* most dominant sense, particularly at short distances‐ up to about one fish length away (Schumacher, Burt de Perera, Thenert, et al., 2016; Schumacher, Burt de Perera, et al., 2017; Schumacher, von der Emde et al., 2017; von der Emde et al., 2010). *Gnathonemus petersii* use pulse‐type EODs to generate their electric fields (von der Emde, 2006). These short, discrete pulses are generated from an electric organ (comprising of modified muscle cells known as electrocytes) found in the caudal peduncle (Bennett, 1971; Lissmann, 1958; Markham, 2013; Moller, 1980; Fig. 2). The firing of these electrocytes is controlled by the pacemaker nucleus in the hindbrain (Bennett, 1971; Markham, 2013). Each EOD produces a three‐dimensional electric field around the fish with a set geometry according to properties of the fish itself (Schwarz, 2000 as cited in von der Emde, 2006; Caputi & Budelli, 2006; von der Emde, 2006; Schumacher, Burt de Perera, von der Emde, et al., 2016). It is an asymmetric dipole field, with a larger pole covering the body anterior to the electric organ, and a smaller pole at the fish's tail (Engelmann et al., 2008; von der Emde, 2006). Objects within the generated electric field distort it and as a consequence the locally perceived EODs. How the electric field is distorted depends on the properties of the object. For example, an object which is an electrical conductor will increase electric field line density, and thus increase the amplitude and distort the waveform of locally perceived EODs opposite the object (von der Emde, 2006; Figs 2 & 3). Specialised tuberous electroreceptors on the skin of the fish (known as mormyromasts) are tuned to detect these voltage pattern changes caused by the object (Bell et al., 1989; Szabo & Wersäll, 1970; von der Emde, 2006; von der Emde & Bleckmann, 1992). The area on the skin that is affected by the object is called the ‘electric image’ (Caputi & Budelli, 1995). The mormyromasts are distributed asymmetrically on the body. The highest density of receptors can be found on the head, particularly in the nasal region (just above the mouth) and on the Schnauzenorgan, their movable chin appendage (Harder, 1968; Hollmann et al., 2008; Pusch et al., 2008; von der Emde & Schwarz, 2002; Fig. 2). Few receptors are found on the sides, and none on the tail (Harder, 1968; Hollmann et al., 2008; Pusch et al., 2008; von der Emde & Schwarz, 2002; Fig. 2). The Schnauzenorgan and nasal region are considered ‘electric foveae’, not only due to the high density of receptors they both have but also because of the morphological, physiological and behavioural adaptations associated with these regions, which likely make them well‐tuned for different aspects of foraging (Amey‐Özel et al., 2012; Bacelo et al., 2008; Hollmann et al., 2008; Pusch et al., 2008; von der Emde et al., 2008).

The electric images produced by the presence of objects are blurred as there is no focussing mechanism akin to that found in vertebrate visual systems (Caputi & Budelli, 2006; von der Emde, 2006; Fig. 3). Nevertheless, this sense provides excellent fine scale spatial information about objects in close proximity to the fish (Schumacher, Burt de Perera, et al., 2017; von der Emde, 2006; von der Emde et al., 2010). *Gnathonemus petersii's* EODs are biphasic as they have two phases, a positive and negative phase (Bell & Russell, 1978; von der Emde, 1993; Fig. 2). Since *G. petersii's* EODs have a fairly stereotyped appearance, but the time between their pulses (known as inter‐discharge intervals, or IDIs) is highly variable, they encode information in a temporal format (Engelmann et al., 2008; Sawtell et al., 2005; von der Emde, 1993). The electrical information is first transmitted to the electrosensory lateral line lobe in the hindbrain, and then sent to the nucleus lateralis in the midbrain for further processing where a somatotopic map of the active electric sense exists (Hollmann et al., 2016).

*Gnathonemus petersii* is also a well‐studied organism for the investigation of so‐called neural corollary discharge mechanisms (e.g. Bell, 1981, 1989; Bell et al., 1997; Bell & Grant, 1989; Fukutomi & Carlson, 2020a; also see recent review Fukutomi & Carlson, 2020b; Requarth et al., 2014; von der Emde & Bell 1996). Such mechanisms can be found in many sensory systems and are generally used to deal with the consequences of motor output on sensory processing in the brain (see reviews: Bell, 1981; Fukutomi & Carlson, 2020b; Poulet & Hedwig, 2007; Sawtell et al., 2005). During active electrolocation, the fish's goal is to detect an object via object‐induced electric field distortions (von der Emde, 2006). However, there can also be other causes for such distortions, for example, self‐generated bending of the fish's body (Bell, 1989). In order to eliminate responses of electroreceptive brain areas to these self‐generated stimuli, these animals have evolved an electric organ corollary discharge (EOCD), which is a neuronal ‘copy’ of the electromotor commands that are sent from motor areas involved in EOD generation to sensory areas involved in processing of electrosensory stimuli (Bell et al., 1983). In addition to eliminating responses to self‐generated stimuli in the neurons of the electrosensory system, it is also used to predict the occurrence of an EOD and to prepare the sensory areas of the brain processing this information, enhancing their response to relevant information (Bell et al., 1997). In this review, we will not be discussing the mechanisms behind corollary discharge in further detail as this topic requires its own dedicated discussion (see Fukutomi & Carlson, 2020b). However, it is important to note that the EOCD is an important prerequisite for mormyrid fish to be able to investigate object properties such as three‐dimensional shape during active electrolocation (Fukutomi & Carlson, 2020b). To do so, an individual will engage in movements around an object (*see active sensing strategies section below*) which involves many bends and stretches of the body (e.g., Toerring & Belbenoit, 1979; Toerring & Moller, 1984). Since the EOCD eliminates the electrosensory consequences of these body movements, an individual can perceive the changes in voltage pattern across the skin produced by an object without interference by unwanted body movements.

## *Gnathonemus petersii's* object recognition abilities with their active electric sense

### Deriving information from a single electric image

We have already established that *G. petersii* primarily use their active electric sense at short range, and since it has high spatial resolution, it is the preferred sense for object recognition (e.g. Gottwald et al., 2018; Schumacher, Burt de Perera, Thenert, et al., 2016; Schumacher, Burt de Perera, von der Emde, et al., 2016; Schumacher, Burt de Perera, et al., 2017; Schumacher, von der Emde, et al., 2017; von der Emde, 2006; von der Emde et al., 2010). Research has shown that different image parameters from a single electric image can be used to work out a whole array of object properties. As a reminder, an electric image refers to the area of skin where there is a change in voltage pattern due to the presence of an object (von der Emde, 2006; Fig. 3).

In mormyrids like *G. petersii*, a typical electric image projected onto the skin surface has a centre‐surround (‘Mexican hat’) profile, with a central region surrounded by a contrasting rim area, with the effects dependent on the electric properties and distance of the object (Caputi et al., 1998; Caputi & Budelli, 2006; von der Emde, 2006; Fig. 3). From the electric image, various parameters can be extracted, such as: EOD waveform modulations, image width, maximal slope, peak amplitude and electric colour (Fig. 3). Using one or more of these parameters, *G. petersii* can detect where an object is located, the object's distance, the size of the object and the object's resistance as well as its capacitance (reviewed by von der Emde, 2006). Electric colour is used to identify objects, in particular prey items (Gottwald et al., 2018).

Distance can be estimated from a single electric image by taking the ratio between the maximal slope and maximum amplitude of the image (von der Emde et al., 1998; Fig. 3). This ratio is dependent only on distance (so long as the rostral slope of the image is used) and not on size, shape and material for most objects (von der Emde et al., 1998). The slope of the electric image is determined by the ‘fuzziness’ of the image's edges, with nearer objects making the electric image appear sharper, while further away objects will make it appear more blurred (von der Emde, 2006; von der Emde et al., 1998).

However, these fish do not experience three‐dimensional objects purely statically, they will usually move around objects in set patterns (Toerring & Belbenoit, 1979; Toerring & Moller, 1984). It is therefore important to identify, characterise and assess these behaviours to determine if these movements might influence perception, and in doing so, help to provide insight on part of the action‐perception cycle (Hofmann, Sanguinetti‐Scheck, Künzel, et al., 2013).

### Active sensing strategies identified in mormyrids can be used to interrogate sensory‐motor links

Several set behaviours (‘probing motor acts or PMAs’) have been identified in mormyrids (including *G. petersii*) during object exploration and are described in the seminal papers of Toerring and Belbenoit (1979), Toerring and Moller (1984) and von der Emde (1992). One such behaviour is chin probing, when an individual (slowly) approaches an object, touches it briefly with their chin appendage (Schnauzenorgan), and then moves away (Toerring & Belbenoit, 1979; Toerring & Moller, 1984; von der Emde, 1992; von der Emde & Fetz, 2007). This occurs most frequently at the start of exploration when there is less familarization with the object (Toerring & Belbenoit, 1979). Backwards swimming is also common when an individual is approaching a novel object (von der Emde, 1992; von der Emde & Fetz, 2007). Stationary probing occurs when a fish approaches an object directly, and stops rapidly once the head is a set distance from the object, often occurring more towards the end of object exploration, and so differing from chin probing (Toerring & Belbenoit, 1979; Toerring & Moller, 1984; von der Emde, 1992; von der Emde & Fetz, 2007). von der Emde (1992) also observed stationary wriggling, where a fish remained stationary by an object but then would initiate whole‐body movements, resulting in the distance between the object and the receptive surface of the fish to alternate. *‘Va‐et‐vient’* is another behaviour, where an individual swims back and forth repeatedly beside an object at a fixed distance, and can occur both radially and laterally (Toerring & Belbenoit, 1979; Toerring & Moller, 1984; von der Emde, 1992; von der Emde & Fetz, 2007). This back and forth motion is often associated with tail probing, where the tail is moved side to side by the object (Toerring & Belbenoit, 1979; von der Emde & Fetz, 2007). Tangential probing occurs when the fish heads directly towards an object, then rapidly changes their direction (Toerring & Belbenoit, 1979; Toerring & Moller, 1984). This differs to lateral probing which involves the fish swimming towards an object, followed by a forward circling movement, where they maintain a set distance from the object (Toerring & Belbenoit, 1979; Toerring & Moller, 1984). These movements were found to coincide with changing EOD activity and so Toerring and Belbenoit (1979) and Toerring and Moller (1984) assumed that they played a role in sensing. Indeed, when individuals were ‘silenced’ so that their electric organs could no longer fire, some of the probing acts were no longer exhibited, further supporting the hypothesis that these stereotyped behaviours are important for sensory acquisition (Toerring & Moller, 1984).

It has been hypothesised that these behaviours could position the fish in a way in which it optimises active electrolocation (Toerring & Moller, 1984; von der Emde, 1992). For example, during chin and stationary probing, the region of the body nearest to the object is the head and Schnauzenorgan (von der Emde, 1992). Since the nasal region and Schnauzenorgan are ‘electric foveae’, these areas would be best tuned to detect even the subtlest of object details and so it would make sense for them to be the first parts of the body to detect the object (Amey‐Özel et al., 2012; Bacelo et al., 2008; Hollmann et al., 2008; Pusch et al., 2008; von der Emde et al., 2008). It is also theorised that these PMAs might induce maximal change in electric current flow generated across an individual's receptive surface, improving extraction of object properties (Toerring & Moller, 1984). This question of how individuals might use a temporal series of electric images (electric flow) facilitated by self‐driven movement is a question that scientists are still trying to answer.

Motion is hypothesised to modify electrosensory flow which might help perception in a number of ways (Hofmann, Sanguinetti‐Scheck, Künzel, et al., 2013): to modify sensory feedback so that it matches with what the neural system is attuned to (Stamper et al., 2012) and to increase the sensory volume of the animal, and in doing so, maximising the chance of detecting objects in the environment, for example, prey (MacIver et al., 2001; Snyder et al., 2007). Experiments undertaken on *G. petersii* have also shown movement to be important for improving sensory acquisition. Hofmann, Sanguinetti‐Scheck, Gómez‐Sena, et al. (2013) found a novel cue for estimating an object's distance, referred to as the temporal slope‐amplitude ratio, which could be extracted from a succession of electric images and required a very limited sensory surface (a single electroreceptor was sufficient). This method was less susceptible to ambiguity than the static measure of distance previously described (Hofmann, Sanguinetti‐Scheck, Gómez‐Sena, et al., 2013; von der Emde et al., 1998). Pedraja et al. (2018) showed that these movements make distance perception by motion parallax possible. Another recent study found that during an object detection task, *G. petersii* learned to adjust their behaviour in a way which seemed to improve the distance at which objects could be detected (Pedraja et al., 2020). Nonetheless, the study of how and why movement modulates sensory flow in weakly electric fish is still in its infancy, with new insights continuing to be made, often combining vital empirical (behavioural) work with theoretical approaches to classify and quantify the behaviour and the effects on sensory flow (see reviews: Engelmann et al., 2021; Hofmann, Sanguinetti‐Scheck, Künzel, et al., 2013). This allows questions relating to sensory‐motor integration to be investigated more comprehensively. One such question is whether movement might help with identifying three‐dimensional object properties that might otherwise be difficult to extract.

### Difficulties with extracting information from a single electric image using shape recognition as an example

Not all object properties can be explained by one or more image parameters within an electric image. Shape is one such example. We know that *G. petersii* can recognise three‐dimensional shapes but it has been difficult to attribute this to known parameters of the electric image (Schumacher, Burt de Perera, von der Emde, et al., 2016; Skeels, 2022; von der Emde, 2004, 2006; von der Emde et al., 2010; von der Emde & Fetz, 2007; von der Emde & Schwarz, 2000, 2002). The shape of an electric image cannot simply be taken as the shape of the object projecting that image, due to distortion relating to the way the electric field is generated and maintained (Caputi & Budelli, 2006; Pusch et al., 2008; von der Emde, 2006).

Past studies have suggested that individuals probably favour the use of geometric features to characterise and discern between objects (Schumacher, Burt de Perera, et al., 2017; von der Emde et al., 2010; von der Emde & Fetz, 2007)—potentially extracting this information by the analysis of a series of electric images across the body surface (Fujita & Kashimori, 2019; Hofmann, Sanguinetti‐Scheck, Künzel, et al., 2013; Schumacher, Burt de Perera, von der Emde, et al., 2016; Skeels, 2022; Skeels et al., 2022). This would likely occur as a result of an individual engaging in directed movements around the object of interest‐ such as the probing motor acts we described earlier (Toerring & Belbenoit, 1979; Toerring & Moller, 1984). This would cause the electric images projected onto the skin to change as the position of the fish shifts in relation to the object (Hofmann, Sanguinetti‐Scheck, Gómez‐Sena, et al., 2013; Hofmann, Sanguinetti‐Scheck, Künzel, et al., 2013) with the magnitude and nature of the modulations reflecting the object's shape (Schumacher, Burt de Perera, von der Emde, et al., 2016). In fact, recent studies have investigated the role of egocentric movement in the extraction of three‐dimensional shape information and have demonstrated its importance for shape recognition (Schumacher, Burt de Perera, von der Emde, et al., 2016; Skeels, 2022; Skeels et al., 2022). The problem of shape recognition described here has provided us with the opportunity to examine how sensory and motor systems might work together to resolve recognition difficulties. It has shown us how self‐driven movement can allow the extraction of shape information that might otherwise be inaccessible to the animal. It also provides a platform to ask further questions on the nature of sensory‐motor integration.

## Conclusions

This review investigates sensory‐motor integration in mormyrids, using *G. petersii* as a model species. *Gnathonemus petersii* have a unique and accessible sensory system and exhibit stereotyped behaviours which make them suitable for studying sensory‐motor interactions. These can be studied simultaneously which is advantageous given that their sensory and motor systems are continuously providing feedback to one another. *Gnathonemus petersii* can use self‐driven motion to actively shape their electrosensory input, enabling them to optimise perception and extract otherwise unaccessible information from their environment. This has helped them to resolve some of the difficulties associated with complex three‐dimensional object recognition using the active electric sense, for example, shape recognition. The ability to detect, recognise and categorise objects accurately is of vital importance for keeping *G. petersii* competitive in their niche, by allowing them to navigate their cluttered environments successfully, find food obscured by substrate and evade predators. This would be challenging without the dynamic feedback of sensory and motor systems. It is likely that this tight relationship has contributed in part to their success in occupying a broad geographical range spanning central and western parts of Africa. Future work should focus on determining the role(s) of other active sensing strategies long described in these fish (and related species) but with no/limited function(s) attributed to them. Behavioural experiments will be foundational for this, as the behaviour represents a solution to a problem that has taken millions of years to refine. Behaviour work will act as a scaffold which other approaches (e.g. neuronal and theoretical) can build off of. Discussing the neural approach to sensory‐motor integration was beyond the scope of this review, but it is an important aspect to consider and requires its own dedicated review. For example, identifying and characterising the neural pathways activated in mormyrids during PMAs in the context of object exploration is vital as this information is currently lacking. Engelmann et al. (2021) provide an excellent review of up‐to‐date studies that examine the neuronal basis that link active electrosensing with spatial learning. Different disciplines can provide novel insights into the same questions, by looking at different levels of complexity. This is beneficial for developing a more comprehensive picture of what is going on. The best approaches to study sensory‐motor integration will therefore likely involve the use of a variety of theoretical and empirical methods. Nonetheless, we advocate the use of behavioural approaches with minimal invasiveness first so that there is a strong base in which to interrogate some of the most fundamental questions in behaviour, such as how exactly does sensory‐motor integration work and how does it benefit the animal?

Insight from this review can be used to interpret other active sensing strategies in animals using different modalities, since some of the fundamental principles are the same. For example, parallels can be drawn between the active sensing strategies of weakly electric fish and those of echolocating bats. Despite the differences in the type of signal produced and medium of transmission, both animal groups produce behaviours that can either improve perception in general or alter sensory flow. These parallels can also be extended to understanding active sensing strategies in passive senses, for example, active vision or active touch. In these situations, animals do not probe the environment with their own signals, but they can move body parts associated with signal detection to improve their interaction with an external stimulus (e.g. insects moving their eyes when visualising the environment and rats exploring a surface with their whiskers). As such, the findings of this review can be applied to understanding active sensing more generally. Investigating the interaction between sensory and motor systems will help us understand how animals have adapted to their habitats.
